# Topical NSAIDs and Oral Acetazolamide for Macular Edema after Uncomplicated Phacoemulsification: Outcome and Predictors of Non-Response

**DOI:** 10.3390/jcm11195537

**Published:** 2022-09-21

**Authors:** Wissam Aljundi, Loay Daas, Yaser Abu Dail, Barbara Käsmann-Kellner, Berthold Seitz, Alaa Din Abdin

**Affiliations:** Department of Ophthalmology, Saarland University Medical Center (UKS), 66421 Homburg, Germany

**Keywords:** postoperative macular edema, nonsteroidal anti-inflammatory eye drops, oral acetazolamide, uncomplicated phacoemulsification, diabetes mellitus, cumulative dissipated energy

## Abstract

Purpose: To investigate the effectiveness of nonsteroidal anti-inflammatory eye drops (NSAIDs) combined with oral acetazolamide for postoperative macular edema (PME) after uncomplicated phacoemulsification (PE) and identify predictors of non-response. Methods: We analyzed data of uncomplicated PE and identified eyes with PME. First-line therapy included topical NSAIDs combined with oral acetazolamide. In the case of non-response, triamcinolone was administered subtenonally. Outcome measures included best-corrected visual acuity (BCVA) and central macular thickness (CMT). Results: 94 eyes out of 9750 uncomplicated PE developed PME, of which 60 eyes were included. Follow-ups occurred 6.4 ± 1.8, 12.5 ± 3.7 and 18.6 ± 6.0 weeks after diagnosis. BCVA and CMT improved significantly in all follow-ups. In total, 40 eyes showed a response to first-line therapy at the first follow-up (G1). The remaining 20 eyes showed no response and required subtenon triamcinolone (G2), of which 11 eyes showed complete regression at the second follow-up and 4 eyes at third follow-up. A further 5 eyes showed no response and required intravitreal injection. Multivariate linear regression model showed that Diabetes mellitus (DM) and increased cumulative dissipated energy (CDE) are predictors of non-response. Conclusion: Topical NSAIDs with acetazolamide resulted in complete regression of PME in 67% of all cases. DM and increased CDE might be considered as predictors of non-response to this treatment.

## 1. Introduction

Cataract is the leading cause of blindness worldwide, with nearly 18 million people estimated to go blind bilaterally because of cataracts. This is nearly half of all causes of blindness due to eye disease worldwide [1].

Postoperative macular edema (PCME) represents a major complication after cataract surgery, which is the most common eye surgery worldwide. Depending on the technique of the cataract surgery and whether the diagnosis was made clinically or by using retinal imaging techniques such as optical coherence tomography (OCT) and fluorescein angiography (FAG), the incidence ranges in general from 1% to 20% [2,3,4].

A major challenge in the treatment of PCME is the lack of large clinical trials on standardized systematic treatment of PCME, which has led to varying non-evidence-based treatment regimens. Based on the pathophysiology, a common “first-line” treatment of PCME consists of a combination of topical nonsteroidal, anti-inflammatory eye drops (NSAIDs) and systemic oral acetazolamide. In the case of no response to first-line therapy, other options such as subtenon or intravitreal injection of triamcinolone acetat (TA), intravitreal dexamethasone implant, intravitreal injection of anti-vascular endothelial growth factor (VEGF) and finally pars plana vitrectomy (PPV) could be considered [5,6].

The purpose of this study was to investigate the effectiveness of topical NSAIDs in combination with oral acetazolamide to treat PCME after uncomplicated PE. In addition, the predictive factors of non-response to this treatment were evaluated.

## 2. Patients and Methods

A retrospective analysis of the cataract surgeries conducted at the Department of Ophthalmology, Saarland University Medical Center (UKS—Homburg/Saar, Germany) between 1 January 2016 and 31 December 2020. 

## 3. Inclusion Criteria

Eyes of patients of both genders with PCME.

Uncomplicated phacoemulsification (PE).

Mean follow-up time of 18 weeks.

## 4. Exclusion Criteria

Extra- or intracapsular cataract extraction (ECCE/ICCE).

Intraoperative complications of any kind including capsular rupture, loss of lens material in vitreous cavity, and iris trauma, whereas intraoperative use of iris retractors due to mydriasis weakness was not an exclusion criterion.

Eyes with concomitant pathologies leading to increased CMT such as uveitis, history of retinal vascular occlusion, history of diabetic macular edema (DME), and history of exudative age-related macular degeneration (AMD).

Previous treatment with any kind of IVI.

Previous antiglaucomatous treatment with prostaglandin analogues in the last 6 months. 

Pregnancy.

The operations were performed by 10 different surgeons classified into two experience categories:

Category 1: ≥500 previous cataract surgeries (five surgeons).

Category 2: <500 previous cataract surgeries (five surgeons).

The baseline was diagnosis of PCME. This was diagnosed if any patient presented with features of decreased vision or any kind of visual disturbances after uncomplicated PE, with evidence of intraretinal fluid in the macula OCT. In addition, a FAG was performed to exclude other potential causes.

**Figure 1:**
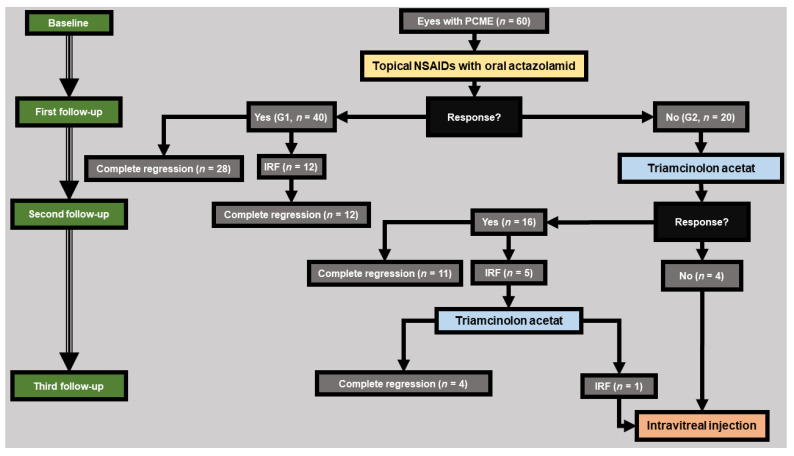
A detailed description of our treatment protocol, as well as the patient distribution in the two groups: **Group 1 (response group G1, *n* = 40)**: eyes that showed a therapeutic response under first-line therapy (NSAIDs eye drops in combination with acetazolamide) at first follow-up. **Group 2 (non-response group G2, *n* = 20)**: eyes that did not show a therapeutic response under first-line therapy and required additional therapy with subtenon triamcinolone acetate. Therapeutic response was defined by a reduction in CMT with an improvement in BCVA. **PCME**: Postoperative cystoid macular edema. **NSAID**: nonsteroidal anti-inflammatory eye drops. **IRF**: intraretinal fluid.

All eyes received a standardized therapy protocol (Figure 1) and were followed up at similar time intervals. First-line therapy included once daily NSAIDs eye drops (nepafenac 3 mg/mL; Nevanac^®^, Novartis, Basel, Switzerland) and twice daily oral acetazolamide (Glaupax^®^ 250 mg, OmniVision GmbH, Puchheim, Germany). At diagnosis, all patients were carefully informed about possible side effects of therapy with acetazolamide (mainly paresthesias, dysgeusia, polyuria, and fatigue).

We defined response to therapy as a reduction in central macular thickness (CMT) with an improvement in best-corrected visual acuity (BCVA).

Therapy was stopped in the case of complete regression of PCME and continued in the case of response with residual intraretinal fluid (IRF). In the case of non-response, a subtenon injection of triamcinolone (Volon A^®^ 40 mg/mL, Dermapharm AG, Grünwald, Germany) was administered. At this point, therapy was discontinued in the case of complete regression. In the case of residual IRF, a further injection was administered. In the case of continued non-response, an intravitreal injection (IVI) was then administered.

Depending on response to first-line therapy at the first follow-up, the eyes were divided into two groups: response group G1 and non-response group G2.

To assess the total ultrasound energy expended at the incision, cumulative dissipated energy (CDE, in %-seconds) was collected from the automatically calculated value displayed on the phacoemulsification device monitor after each surgery. This is typically calculated using the following formula:

Total CDE = (longitudinal time × average longitudinal power) + (torsional time × 0.4 × average torsional amplitude) [7].

A variety of possible general or intraoperative risk factors or causes of non-response to first-line therapy were assessed and compared between the two groups (listed in Table 1).

## 5. Primary Outcome Measures

CMT, in µm, measured using Spectral Domain Optical Coherence Tomography (Spectralis SD-OCT; Heidelberg Engineering, Heidelberg, Germany). The volumetric retinal scans of fovea consisted of 19 parallel B-scans with a spacing of 240 µm and a pattern size of 20 × 15°, automatic real-time repetition and Early Treatment Diabetic Retinopathy Study (ETDRS) map centered on the fovea.

BCVA, in logMAR.

## 6. Statistical Analysis

Data were collected with Microsoft Excel 2010 (Microsoft Corporation, Redmond, WA, USA) and analyzed with SPSS version 26 (SPSS Inc., Chicago, IL, USA).

We used the Mann–Whitney U-Test for not normally distributed variables and *t*-test for normally distributed. For the comparison between frequencies we used the Chi-square (χ^2^) test. To identify potential predictors of non-response, we performed a multivariate linear regression analysis. *p* values < 0.05 were considered statistically significant.

## 7. Results

A total of 9750 uncomplicated PE were performed in our department between 1 January 2016 and 31 December 2020. A total of 94 eyes developed a PCME, of which we included 60 eyes with PCME matching our criteria. The remaining 34 eyes were excluded from this study for following preoperative reasons: history of uveitis (*n* = 2), history of retinal occlusion (*n* = 11), history DME (*n* = 9) and history of exudative AMD (*n* = 12).

The incidence of PCME after uncomplicated PE and regarding our criteria during this period was 0.95%.

The preoperative characteristics of all eyes and of both groups are shown in Table 1.

The observation time started with the diagnosis of PCME, which occurred **6.5 ± 3.9** weeks postoperatively. The first follow-up after the diagnosis of PCME took place after **6.4 ± 1.8** weeks, the second after **12.5 ± 3.7** weeks and the third after **18.6 ± 6.0** weeks.

At the first follow-up, a response to first-line therapy was observed in 40 eyes (G1), of which 28 eyes showed complete regression and 12 eyes showed residual IRF and continued to receive therapy and showed a complete regression at the second follow-up. The remaining 20 eyes showed no response to first-line therapy and required additional therapy with subtenon triamcinolone (G2).

At the second follow-up, response to second-line therapy was observed in 16 eyes in G2, of which 11 eyes showed complete regression and 5 eyes had residual IRF and received a second triamcinolone subtenon injection. The remaining 4 eyes of G2 showed no response and required intravitreal injection therapy. 

At the third follow-up, 4 eyes showed complete regression after the second subtenon triamcinolone injection whereas one eye showed no response and required intravitreal injection. A total of 5 eyes received intravitreal injection therapy: 3 eyes received Ranibizumab (Lucentis 0.5 mg/0.05 mL; Novartis, Nuremberg, Germany) and 2 eyes received a Dexamethasone implant (Ozurdex 0.7 mg; Allergan, Inc., Frankfurt, Germany).

A detailed description of our treatment protocol, as well as the patient distribution in the two groups is shown in Figure 1.

Regarding the entire cohort, the preoperative CMT (in µm) was 301 ± 51. CMT decreased form 483 ± 123 at baseline to 368 ± 65 at first follow-up (*p* < 0.01), to 354 ± 56 at second follow-up (*p* < 0.01) and to 345 ± 57 after third follow-up (*p* < 0.01).

There was no significant difference in CMT between the two groups preoperatively (*p* = 0.83) and at baseline (*p* = 0.29). However, there was a significant difference in CMT at all follow-ups (*p* < 0.01).

Regarding the entire cohort, the preoperative BCVA (in logMAR) was 0.38 ± 0.46. BCVA improved from 0.40 ± 0.27 at baseline to 0.22 ± 0.21 at first follow-up (*p* < 0.01), to 0.23 ± 0.22 at second follow-up (*p* < 0.01) and to 0.22 ± 0.23 at third follow-up (*p* < 0.01).

There was no significant difference in BCVA between the two groups preoperatively (*p* = 0.59), at baseline (*p* = 0.53) and at the third follow-up (*p* = 0.21). However, there was a significant difference in BCVA at the first and second follow-up (*p* = 0.02 and *p* = 0.01 respectively).

The change in CMT (in µm) and in BCVA (in logMAR) is shown in Table 2 and Table 3.

The analysis of patient-related, ocular or intraoperative data to identify potential predictors of non-response is shown in Table 4. The performed multivariate linear regression analysis showed that a history of diabetes mellitus (DM) (*p* = 0.03) as well as an increased CDE (*p* = 0.01) significantly contribute as predictors of non-response to first line-therapy.

No relevant adverse events were reported up to the last follow-up.

**Figure 2:**
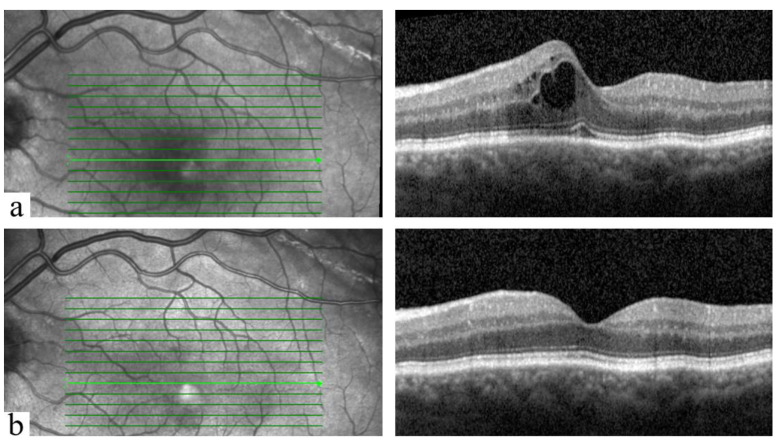
An example of OCT findings of an included patient of group 1 (male, 69 years old): (**a**) At the diagnosis of PCME. Central macular thickness (CMT) was 425 µm. (**b**) After treatment with once daily NSAIDs eye drops (nepafenac 3 mg/mL; Nevanac^®^) and oral acetazolamide (Glaupax^®^ 250 mg). CMT was 311 µm. PCME occurred 33 days postoperatively and resolved at the first follow-up after 28 days.

An example of OCT findings at diagnosis and after treatment of PCME is shown in Figure 2.

## 8. Discussion

The incidence of PCME was examined in a large study in 2016 on more than 81,000 eyes and was estimated to be approximately 1–2% after uncomplicated PE, which is related to the incidence we found in our study (0.95%) [8,9].

Many previously known risk factors for the development of PCME are listed in Table 5 [8,9].

The pathogenesis of PCME remains unclear. However, postoperative inflammation remains the main hypothesis. Many inflammatory pathways, including lipoxygenase and cyclooxygenase, are activated after the surgical manipulation due to increased inflammatory mediators produced from the uveal tissue, such as prostaglandins and leukotrins. The increased concentration of these mediators in the vitreous cavity leads to increased permeability of the perifoveal capillaries, which later results in the formation of the PCME. Yet it remains unclear why this effect only involves the perifoveal area and not the whole retina. Based on this, the aim of the treatment of PCME was to inhibit inflammation and to reduce extracellular fluid [3,6,8,9].

A common first-line therapy includes NSAIDs eye drops often in combination with oral acetazolamide. The often-used NSAID eye drops for the treatment of PCME, nepafenac, can be transformed to the more active metabolite amfenac by intraocular hydrolases in vascular ocular tissues and act as a potent inhibitor of COX-1 and COX-2 activity. Acetazolamide enhances the pumping action of the retinal pigment epithelium by acting on carbonic anhydrase. However, its mechanism of action in treatment of intraretinal macular edema (i.e., in uveitis) is still unclear [11,12,13,14].

There is some evidence in the literature that NSAID eye drops are effective for prophylaxis and treatment of PCME [9,10,11,12,15]. The use of acetazolamide was first investigated in the 1980s. Several case series reported a favorable effect of acetazolamide in the treatment of chronic macular edema associated with various diseases such as retinitis pigmentosa and uveitis [14,16,17]. However, the use of acetazolamide to treat PCME lacks strong evidence or confirming studies in the literature and is based only on several previously published case reports [18,19].

Over time, cataract surgery evolved in terms of technique, incision, surgical instruments and devices, minimizing the surgical manipulation, which was considered one of the main risk factors for PCME, and thus PCME became even rarer. 

However, this also resulted in the lack of major studies regarding the appropriate treatment. For this reason, the use of current regimens is based on the pathophysiologic knowledge and lacks support from large trials. 

A recent study has examined the use of topical NSAIDs and intravitreal or peribulbar steroids in 33 eyes [20]. PCME occurred after 4 weeks postoperatively. Topical NSAIDs eye drops were solely used as first-line therapy and resulted in complete regression of PCME in 19 of the 33 eyes studied (57%). A Dexamethasone implant was the second-line therapy and was performed in only 7 eyes, as the remaining 6 eyes refused this therapy and received peribulbar triamcinolone instead [20].

Unlike our study, the rate of complicated cataract surgery was 51.5%. It would be interesting to know how many of the remaining 14 eyes, which showed no response to topical NSAIDs, underwent complicated cataract surgery, and whether there was a correlation between complicated cataract surgery and non-response to first-line therapy.

Our study included 60 eyes with PCME after uncomplicated PE. Considering intraoperative complications as an exclusion criterion, it helped to reduce the impact of what is widely considered the most important risk factor for the development and severity of often refractory PCME on non-response to first-line therapy and gave us a realistic understanding of PCME treatment in real life. Especially the fact that PE is usually uncomplicated (worldwide overall intraoperative complication rate is about 6–7%) [10,21,22].

The aim of our study was to investigate the effectiveness of a commonly used therapeutic regimen for the treatment of PCME. An effective regimen may reduce the duration of PCME and spare macular function and morphology. Regardless of what would be considered as second or even third-line therapy, the use of NSAIDs eye drops as first-line therapy for the treatment of PCME has been agreed upon in the literature [8,9,12,15]. This shows the importance of our study to identify “high-risk” eyes based on preoperative or intraoperative risk factors and eventually consider a more aggressive treatment and/or shorter follow-ups to detect non-response at an early stage and modify therapy accordingly.

We found that prevalence of DM, as well as increased CDE intraoperatively were significant risk factors for non-response to therapy.

DM results in an impaired blood–retinal barrier, increased inflammatory molecules, increased vascular permeability and many other retinal pathologies. Furthermore, it is well known in the literature that patients with DM have an increased risk of PCME [8,9,23,24].

In 1999, Ferrari et al. found that the use of high phaco energy had led to disruption of the blood–retinal barrier and development of a PCME. Similarly, we found that increased CDE had led more frequently to non-response to first-line therapy. This could be explained by the increased intraoperative trauma-induced vasodilated molecules resulting in a more severe PCME [25,26].

This supports the consideration that PCME in these three cases is potentially more challenging and could require a supportive initial therapy with subtenon triamcinolon.

Previously published papers found that there is moderate to poor correlation of CMT and BCVA. Furthermore, PCME is mainly characterized by SRF, loss of foveal depression, and cystic hyporeflective lesions. Moreover, the visual impairment associated with PCME is due to the increase in CMT and not to permanent retinal photoreceptor damage (as in other diseases such as AMD) [27,28]. This could possibly explain our results, as we found that there was no significant difference in BCVA between responders’ and non-responders’ eyes at the last follow-up, with, however, a significant difference in CMT.

In summary, topical NSAID and acetazolamide resulted in a complete regression of PCME after uncomplicated PE in 67% of all cases after 6-12 weeks. DM and increased CDE might be considered as predictive factors associated with non-response to this therapy.

### 8.1. Main Limitations of This Study

The main potential limitations of our study were the retrospective nature of the work, a relatively small population from a single medical center. 

In this study, we assessed visual changes using best-corrected visual acuity (BCVA, logMAR), which may not have detected subtle visual changes compared with other well-calibrated visual function tests such as log contrast sensitivity.

### 8.2. Value Statement

#### 8.2.1. What Was Known?

Postoperative cystoid macular edema (PCME) is one of the most important and common complications of cataract surgery, which has no standardized therapeutic regimen and is commonly treated with a combination therapy of NSAIDs eye drops and oral acetazolamide.

Over time, cataract surgery evolved in terms of technique, incision and surgical instruments and devices, and PCME became rarer. Nevertheless, this also led to the lack of major studies on the appropriate treatment.

The use of this therapy is based on the pathophysiology of PCME, and its efficacy has not been studied. In addition, the influence of patient-related preoperative or intraoperative factors on the response to therapy has not been investigated.

#### 8.2.2. What This Paper Adds?

This paper suggests that NSAID eye drops in combination with acetazolamide have an efficacy of 67% in achieving complete regression of PCME after uncomplicated phacoemulsification. Diabetes mellitus, as well as increased cumulative dissipated energy may be considered as predictive factors for non-response to this therapy and therefore may require supportive initial therapy with subtenon triamcinolone.

## Figures and Tables

**Table 1:** Preoperative characteristics of the eyes studied.

Preoperative Characteristics of the Eyes Studied			
	Response Group (G1)	Non-Response Group (G2)	*p*-Value (G1 vs. G2)
**Number of eyes**	40	20	
**Age (in years)**	71 ± 10	70 ± 17	0.61 †
**Gender (male/female) %**	60/40	45/55	0.43 *
**Eye (right/left) %**	47/53	55/45	0.01 †
**Preoperative CMT (in µm)**	303 ± 37	297 ± 72	0.83 ‡
**Preoperative BCVA (in logMAR)**	0.43 ± 0.54	0.27 ± 0.22	0.59 ‡
**Eye length (in mm)**	23.4 ± 1.3	23.7 ± 2	0.85 †

†: student’s *t*-test, *: Chi-square (χ^2^) test, ‡: Mann–Whitney U-Test. CMT: central macular thickness. BCVA: best-corrected visual acuity. **Group 1 (response group G1, *n* = 40)**: eyes that showed a therapeutic response under first-line therapy (NSAIDs eye drops in combination with acetazolamide) at first follow-up. **Group 2 (non-response group G2, *n* = 20)**: eyes that did not show a therapeutic response under first-line therapy and required additional therapy with subtenon triamcinolone acetate.

**Table 2:** The change in central macular thickness (CMT, in µm) during the study.

The Change in Central Macular Thickness (CMT, in µm) during the Study			
	Response Group (G1)	Non-Response Group (G2)	*p*-Value (G1 vs. G2)
**Preoperative CMT**	303 ± 37	297 ± 72	0.83 ‡
**CMT at baseline**	479 ± 111	491 ± 146	0.29 ‡
**CMT after 6.4 ± 1.8 weeks**	337 ± 31	429 ± 72	***<0.01* ‡**
**CMT after 12.5 ± 3.7 weeks**	330 ± 39	409 ± 50	***<0.01* ‡**
**CMT after 18.6 ± 6.0 weeks**	319 ± 36	366 ± 65	***<0.01* ‡**

‡: Mann–Whitney U-Test. CMT: central macular thickness. **Group 1 (response group G1, *n* = 40)**: eyes that showed a therapeutic response under first-line therapy (NSAIDs eye drops in combination with acetazolamide) at first follow-up. **Group 2 (non-response group G2, *n* = 20)**: eyes that did not show a therapeutic response under first-line therapy and required additional therapy with subtenon triamcinolone acetate.

**Table 3:** The change in best-corrected visual acuity (BCVA, in logMAR) during the study.

The Change in Best-Corrected Visual Acuity (BCVA, in logMAR) during the Study			
	Response Group (G1)	Non-Response Group (G2)	*p*-Value (G1 vs. G2)
**Preoperative BCVA**	0.43 ± 0.54	0.27 ± 0.22	0.59 ‡
**BCVA at baseline**	0.39 ± 0.23	0.41 ± 0.33	0.53 ‡
**BCVA after 6.4 ± 1.8 weeks**	0.20 ± 0.21	0.26 ± 0.20	***0.02* ‡**
**BCVA after 12.5 ± 3.7 weeks**	0.19 ± 0.22	0.29 ± 0.20	***0.01* ‡**
**BCVA after 18.6 ± 6.0 weeks**	0.17 ± 0.21	0.25 ± 0.24	0.21 ‡

‡: Mann–Whitney U-Test. BCVA: best-corrected visual acuity. **Group 1 (response group G1, *n* = 40)**: eyes that showed a therapeutic response under first-line therapy (NSAIDs eye drops in combination with acetazolamide) at first follow-up. **Group 2 (non-response group G2, *n* = 20)**: eyes that did not show a therapeutic response under first-line therapy and required additional therapy with subtenon triamcinolone acetate.

**Table 4:** The analysis of patient-related, ocular or intraoperative possible risk factors for non-response to therapy with NSAID eye drops and acetazolamide.

Analysis of Possible Risk Factors of Non-Response to First-Line Therapy							
	Response Group (G1)	Non-Response Group (G2)	Regression Analysis				
			Unstandardized Coefficients		Standardized Coefficients	*t*	*p*-Value
			B	Std. Error	Beta		
**Age (in years)**	71.3 ± 10.5	70.8 ± 17.6	0.006	0.008	0.163	0.733	0.46
**Eye length (in mm)**	23.4 ± 1.3	23.7 ± 2	−0.045	0.056	−0.154	−0.812	0.42
**Gender (M/F) %**	60/40	45/55	0.088	0.158	0.094	0.557	0.58
**History of hypertension**	75%	85%	−0.173	0.180	−0.163	−0.963	0.34
**History of diabetes mellitus**	10%	40%	0.383	0.175	0.336	2.189	***0.03***
**History of hypothyroidism**	32%	35%	0.180	0.178	0.180	1.008	0.32
**History of glaucoma**	32.5%	35%	−0.120	0.277	−0.079	−0.433	0.66
**Preoperative cornea guttata**	5%	5%	−0.259	0.366	−0.631	−0.709	0.48
**Myopia per magna**	5%	15%	0.137	0.310	0.083	0.442	0.66
**Pseudoexfoliation syndrome**	12.5%	45%	0.146	0.168	0.128	0.870	0.39
**Expert surgeon**	72.5%	70%	−0.085	0.143	−0.084	−0.597	0.55
**Intraoperative use of viscoat**	12.5%	5%	−0.172	0.304	−0.113	−0.565	0.57
**Intraoperative use of epinephrin**	32.5%	15%	−0.090	0.176	−0.087	−0.514	0.61
**Intraoperative use of iris retractors**	12.5%	20%	0.005	0.210	0.004	0.024	0.98
**Intraoperative use of Miochol**	30%	25%	0.006	0.006	1.052	1.106	0.27
**Operation duration (in minutes)**	16.2 ± 11	14.3 ± 7.6	−0.003	0.007	−0.071	−0.511	0.61
**Cumulative dissipated energy (CDE, %-seconds)**	4.9 ± 5.6	10.4 ± 5.9	0.027	0.011	0.364	2.468	***0.01***

**Group 1 (response group G1, *n* = 40)**: eyes that showed a therapeutic response under first-line therapy (NSAIDs eye drops in combination with acetazolamide) at first follow-up. **Group 2 (non-response group G2, *n* = 20)**: eyes that did not show a therapeutic response under first-line therapy and required additional therapy with subtenon triamcinolone acetate.

**Table 5:** Known risk factors for the development of PCME [8,9,10].

General Risk Factors	Intraoperative Risk Factors	Ocular Risk Factors
Diabetes mellitus	Capsular rupture	A history of vascular occlusion
Arterial hypertension	Loss of lens material into vitreous cavity	A history of uveitis in all forms
Male gender	Iris trauma	Epiretinal membrane
Age	Use of iris retractors	Previous retinal detachment
	Surgical degree of difficulty	

## Data Availability

Data will be available upon request.
